# Mechanical Properties and Freeze–Thaw Cycling Degradation of Loess Improved with an Ionic Curing Agent and Cement Composite

**DOI:** 10.3390/ma19061242

**Published:** 2026-03-21

**Authors:** Xingwei Wang, Jiandong Li, Xu Wang, Baiwei Li, Yanjie Zhang, Zhen Zuo

**Affiliations:** 1 School of Civil Engineering, Lanzhou Jiaotong University, Lanzhou 730070, China; wangxingwei202208@163.com (X.W.); 18137363162@163.com (B.L.);; 2 National and Local Joint Engineering Laboratory for Disaster Prevention and Control in Road and Bridge Engineering, Lanzhou 730070, China

**Keywords:** improved loess, ionic curing agent, cement stabilization, freeze-thaw durability, NMR pore size distribution

## Abstract

To address the engineering problems of high cement content, high brittleness, and weak frost resistance of cement-improved loess in the seasonal frozen soil area of Northwest China, F1 ion curing agent (F1) and cement composite improved loess (FCIL) were used in this paper. Through unconfined compressive (UC) strength tests, consolidated undrained (CU) triaxial shear tests, and microscopic pore characteristics analysis, the mechanical properties, freeze–thaw cycle deterioration law, and microscopic pore structure of FCIL were studied. The effects of cement content (*C*_c_), F1 dosage (*C*_F_), number of freeze–thaw cycles (*N*_F-T_), and confining pressure (*σ*_3_) on the strength, deformation behavior, and pore characteristics of FCIL were analyzed. The synergistic improvement mechanism of FCIL, as well as the freeze–thaw damage mechanism, was elucidated. The results show that *C*_c_ is the primary factor controlling the strength of improved loess. The incorporation of F1 can further increase UCS and markedly enhance the failure strain (*ε*_f_), thereby achieving simultaneous improvements in strength and ductility. An appropriate mix proportion was identified as *C*_F_ = 0.2 L/m^3^ and *C*_c_ = 6%. After 7 d curing, FCIL exhibited a UCS of 1.35 MPa, a cohesion (*c*) of 205 kPa, an internal friction angle (*φ*) of 36.2°, and *ε*_f_ 1.8 times that of loess improved with *C*_c_ = 6% cement alone. CU triaxial shear tests indicate that, under all tested conditions, the stress–strain responses of FCIL exhibit σ3-sensitive strain-softening behavior. As *C*_c_ and *σ*_3_ increase, triaxial peak strength (*q*_max_) and secant modulus (*E*_50_) increase significantly. Compared with natural loess (NL), FCIL shows a markedly lower porosity (*n*), a substantial increase in the proportion of micropores, and reductions in medium and small pores. After multiple freeze–thaw cycles, the evolution of the pore structure is effectively restrained. This indicates that the combined use of F1 and cement promotes the formation of a dense layered stacking structure, significantly improves the microscopic pore-size distribution, and enhances the mechanical performance of loess under freeze–thaw environments.

## 1. Introduction

Collapsible loess is widely distributed in the arid and semi-arid regions of Northwest China. It exhibits unfavorable engineering characteristics, including weak cementation, pronounced collapsibility, high water sensitivity, a well-developed pore structure, and poor particle gradation. Upon wetting, engineering problems such as slope failures, embankment seepage, foundation cracking, and differential settlement often occur, severely compromising the safety and serviceability of infrastructure in loess areas [1,2]. Therefore, when loess is used as construction fill or as an engineering geomaterial, it must be treated to ensure the safety and stability of engineering structures. Traditional cementitious binders, such as cement, lime, and fly ash, are currently the most widely used stabilizers for loess; their hydration products can fill pores and bind soil particles, thereby significantly improving the strength and water stability of loess. Owing to their high stabilization efficiency and simple, rapid construction procedures, these materials have been widely applied in the construction of highways, railways, airports, and other infrastructure in loess regions [3,4]. However, engineering practice has shown that cement-stabilized loess has shortcomings, such as high brittleness and poor deformation compatibility [5,6]. Under freeze–thaw cycling, microcracks readily develop, and the cemented structure can be damaged, resulting in a pronounced reduction in the strength of cement-stabilized loess as *N*_F-T_ increases [7,8]. In addition, the production and use of traditional cementitious materials (e.g., cement) are associated with high energy consumption and substantial environmental impacts, which are inconsistent with the goals of green and low-carbon construction and energy conservation and emission reduction in engineering practice [9,10].

In view of these issues, engineering researchers have gradually shifted their focus to new soil stabilizers that are green, low-energy, and cost-effective. Among them, ionic curing agents have attracted widespread attention due to their low cost, low dosage, favorable strengthening efficiency, convenient construction, and environmental friendliness [11]. Li et al. [12] observed that the F1 can disrupt the electric double-layer structure through ion exchange, promote the flocculation and aggregation of fine soil particles, and effectively improve the physical and mechanical properties of loess. In the field of loess stabilization, various composite reinforcement schemes have also been systematically investigated and have demonstrated remarkable effectiveness. Yang et al. [13] found that the combined use of cement and silica fume could markedly increase the shear strength of loess: the 28 d shear strength of loess stabilized with 3% cement and 10% silica fume reached 2255 kPa, which was 34.9% higher than that of natural loess. Xue et al. [14] and Wang et al. [15] determined the optimal mix proportion for loess stabilization using composite materials, including cement, SCA-2 curing agent, polypropylene fiber, fly ash, and solid waste from power plants, based on orthogonal tests. Their results showed that cementitious products generated through the synergistic action of multiple components can fill pores and bond soil particles, and the reinforcing effect of fibers further optimizes the soil structure, thereby significantly improving the strength, water stability, and durability of loess. However, such composite systems are sensitive to the dosage ratio and activity of each component, and poor stabilization performance may occur due to segregation, agglomeration, or insufficient reactions. Wang et al. [16] adopted a composite stabilizer of 10% cement and 0.5% calcium lignosulfonate and found that, compared with natural loess, the UCS increased by 13-fold and *E*_50_ increased by 9-fold after 28 d of curing; moreover, after 15 freeze–thaw cycles, the strength loss rate decreased markedly from 72% to 28%. Ma et al. [17] observed that when soil was stabilized with 0.02% CG-2 curing agent and 6% cement, the UCS reached 3.6 MPa, which was 71% higher than that of cement-stabilized soil, indicating that cement consumption can be effectively reduced. Niu et al. [18] found that the combination of 0.3% polypropylene fiber and 2% cement significantly enhanced the shear strength and freeze–thaw durability of loess; when the fiber length was 12 mm, the triaxial shear strength reached 1260 kPa, representing a 16-fold increase compared with untreated loess, and the additional reduction in triaxial strength from 12 to 15 cycles was only 1.9%. Guo et al. [4] found that nano-SiO_2_ could significantly increase the strength parameters of loess stabilized with 2% cement; when the nano-SiO_2_ content increased from 0.2% to 1.5%, the triaxial strength increased from 921 to 1705 kPa at 7 d, and from 1462 to 2491 kPa at 28 d. Ghadakpour et al. [19] reported that cement treatment markedly improves the mechanical behavior of loess and clayey soil, but at the same time, it may shift the material response from ductile to brittle and change the corresponding failure mode. Their study indicates that, although cement is effective in enhancing strength, excessive reliance on cement may adversely affect deformation characteristics.

Axel et al. [20] reported that cement generates hydration products through hydration reactions, including three-dimensional network-like calcium silicate hydrate (C-S-H) and calcium aluminate hydrate (C-A-H) gels, as well as ettringite (AFt). These products fill interparticle pores, promote the cementation of soil particles, and form network- or honeycomb-like spatial structures, thereby significantly improving the strength of stabilized soil. Yuan et al. [21] found, based on microstructural analyses, that cementitious products generated by lime reactions can form chemical cementation structures between loess particles, while the addition of polypropylene fibers provides physical reinforcement through fiber bridging and pull-out resistance, significantly enhancing loess strength and inhibiting crack development. Using SEM observations, Gu et al. [22] revealed the hydration process and microstructural evolution of loess stabilized with a cement–phosphogypsum–fly ash–quicklime composite system, and clarified the stage-dependent strengthening mechanisms of different components in the synergistic reinforcement. Based on Scanning Electron Microscopy (SEM), Nuclear Magnetic Resonance (NMR), and X-ray Diffraction (XRD) analyses, Xu et al. [23] observed that cement-stabilized loess undergoes hydration and erosion reactions under acid-rain environments, leading to the development of small pores into medium and large pores. Ma et al. [24] investigated the deterioration mechanism of loess stabilized with a CG-2 curing agent–cement system under combined wetting–drying and freeze–thaw actions, and found that strength and durability can be effectively improved by reducing porosity, enhancing interparticle cementation, and improving particle contact and arrangement. Tchakalova et al. [25] investigated the combined stabilization of loess from northern Bulgaria using cement and natural zeolite. Based on SEM observations, they found that the cement–zeolite system could effectively promote the formation of cementitious products and significantly densify the microstructure during curing, thereby reducing interparticle pores and enhancing the integrity of the soil skeleton.

Overall, existing studies indicate that composite stabilization of loess can mitigate the drawbacks of single additives, and the synergistic effect can significantly enhance the improvement efficiency. To investigate the synergistic reinforcement between a conventional cementitious binder and an ionic curing agent, this study applies cement and the F1 ionic curing agent to stabilize loess. An engineering-feasible mix proportion for improved loess was first identified through UC tests. Subsequently, based on the selected mix, CU triaxial compression tests after freeze–thaw cycling were performed to reveal the freeze–thaw induced changes in the stress–strain behavior of FCIL and its degradation characteristics. In addition, microstructural tests were conducted to elucidate the synergistic reinforcement mechanism by relating the macroscopic mechanical response to microstructural and pore-structure evolution under different *C*_c_ and *N*_F-T_ conditions. The aim is to leverage the advantages of both additives to enhance the strength and stability of improved loess under freeze–thaw cycles while reducing cement consumption, thereby providing a basis for low-cement, freeze–thaw-durable improvement of loess subgrade materials in cold regions.

## 2. Materials and Methods

### 2.1. Materials

The NL used in this study was Q_3_ Malan loess collected in the Lanzhou area. It is dominated by silt-sized particles, and the sampling depth ranged from 50 to 200 cm. The soil samples were yellowish-brown and exhibited unfavorable engineering properties, including well-developed pore structures, low strength, and poor water stability. The F1 was colorless or pale yellow at room temperature, and its main components were a sulfonated acrylic polymer and various acidic surfactants [26] (TerraSmart Company, Hong Kong, China). The cement used in the tests was 42.5-grade ordinary Portland cement(Fengcheng Conch Cement Co., Ltd., Fengcheng, China), and the basic physical parameters of the test materials are listed in Table 1.

### 2.2. Test Scheme Design and Sample Preparation

(1) Sample preparation

**Figure 1:**
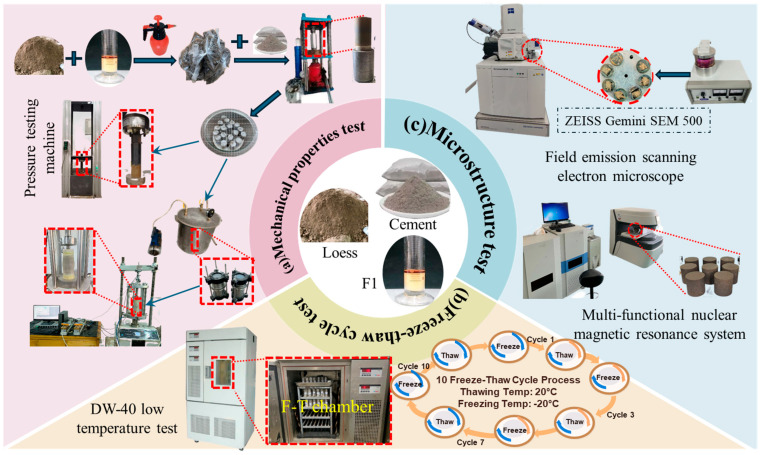
Test design and steps.

After air-drying and crushing, the NL was sieved through a 2 mm sieve, and then uniformly mixed with diluted F1 (dilution ratio: 1:200) according to the prescribed volume ratio, with the soil water content controlled at the optimum moisture content. The mixture was subsequently sealed and left to stand for 24 h to ensure a uniform moisture equilibration. Cement was added at the designed mass fraction 1 h before specimen molding and mixed thoroughly. The degree of compaction was controlled at 95%, and cylindrical specimens for triaxial tests with a diameter of 50 mm and a height of 100 mm were prepared in the specimen mold in three layers. After demolding, the specimens were cured under standard curing conditions for 7 d before subsequent tests; the detailed specimen preparation procedure is shown in Figure 1a. Because the research objectives differ among the various test programs, the curing agent mix proportions adopted in different tests are not identical. The specific mix proportions are provided in the relevant experimental subsections and in Table 2.

(2) Mechanical properties test

The mechanical properties of FCIL were evaluated using unconfined compressive (UC) strength tests and consolidated undrained (CU) triaxial shear tests. The UC tests were performed on a universal testing machine (CMT5504 50 kN microcomputer-controlled electronic universal testing machine, Taizhou Shoufeng Instrument Co., Ltd., Taizhou, China) to compare the strength improvement of loess under different combinations of *C*_F_ and *C*_c_, based on which an appropriate mix proportion of FCIL was determined. The UC tests were conducted on the cement-only group and the cement–F1 jointly improved groups with different F1 dosages. The UC tests were conducted under displacement control at a loading rate of 1 mm/min. After reaching the peak stress, loading was continued, and the test was terminated when the axial strain increased by an additional 3% beyond the strain corresponding to the peak stress. The CU triaxial shear tests were conducted using a fully automated triaxial loading system (TAS-LF fully automated triaxial system, Zhejiang GEO Technology Co., Ltd., Jiaxing, China), and the detailed procedures are shown in Figure 1a. The specimen mix proportions for the CU triaxial shear tests were designed by fixing the F1 dosage (*C*_F_ = 0.2 L/m^3^) while varying the cement content (*C*_c_ = 5%, 6%, 7%, and 8%) to investigate the influence of cement content on the shear behavior of FCIL. Before testing, the specimens were vacuum-saturated, and confining pressure was applied according to the predefined program. During the consolidation stage, the confining pressure was set equal to the test confining pressure. Preliminary tests indicated that the volumetric change became essentially stable after 2 h; therefore, specimens were consolidated for 2 h, and then shearing was initiated under the same confining pressure. The shear rate (*v*) was set to 0.5 mm/min, and the deviator stress–axial strain data were recorded in real time. The test was terminated when the axial strain reached 10%, and the strength and deformation parameters of FCIL were derived from the stress–strain curves. The specific test parameter settings are listed in Table 2.

(3) Freeze–Thaw cycle tests

The freeze–thaw cycle tests were carried out in a DW-40 freeze–thaw chamber (Dongguan Lituo Testing Instrument Co., Ltd., Dongguan, China). After the specimens with the selected optimal mix proportion were cured to the specified age, they were sealed with plastic film and then placed in the freeze–thaw chamber. One complete freeze–thaw cycle consisted of 24 h at −20 °C followed by 24 h at 20 °C, as shown in Figure 1b. After the preset number of freeze–thaw cycles was reached, CU triaxial shear tests were conducted in a timely manner to investigate the effects of freeze–thaw cycling on the mechanical properties of FCIL.

(4) Microstructure test

As shown in Figure 1c, the microstructural characterization of FCIL included NMR tests and SEM observations. For the SEM and NMR tests, natural loess was additionally included as an untreated reference sample. The selected test conditions are listed in Table 2. The NMR tests were performed on vacuum-saturated cylindrical specimens with diameters of 50 mm and heights of 50 mm (MacroMR12-150H-I nuclear magnetic resonance analyzer, Niumag Analytical Instrument Co., Ltd., Suzhou, Jiangsu, China) to obtain the pore-size distribution and the volume fraction of pores at different scales. Prior to the NMR measurements, specimens were vacuum-saturated under a vacuum degree of approximately −0.09 MPa for 2 h to enhance the degree of saturation and facilitate water filling of the pore space. The effects of different *C*_c_ and *N*_F-T_ on the pore structure were then analyzed. For the SEM tests, small block samples were taken from triaxial specimens under different conditions. Before SEM observation, the specimens were oven-dried at a low temperature and then gold-coated for 6 min. SEM images were acquired at magnifications ranging from 1000× to 5000× (ZEISS Gemini SEM 500, Carl Zeiss AG, Oberkochen, Germany). SEM observations were used to examine particle morphology, cementation products, and crack development in FCIL. These observations help clarify the synergistic improvement mechanism of F1 and cement and the freeze–thaw deterioration at the microscopic scale.

## 3. Results and Discussion

### 3.1. Effect of C_c_ and C_F_ on UCS

**Figure 2:**
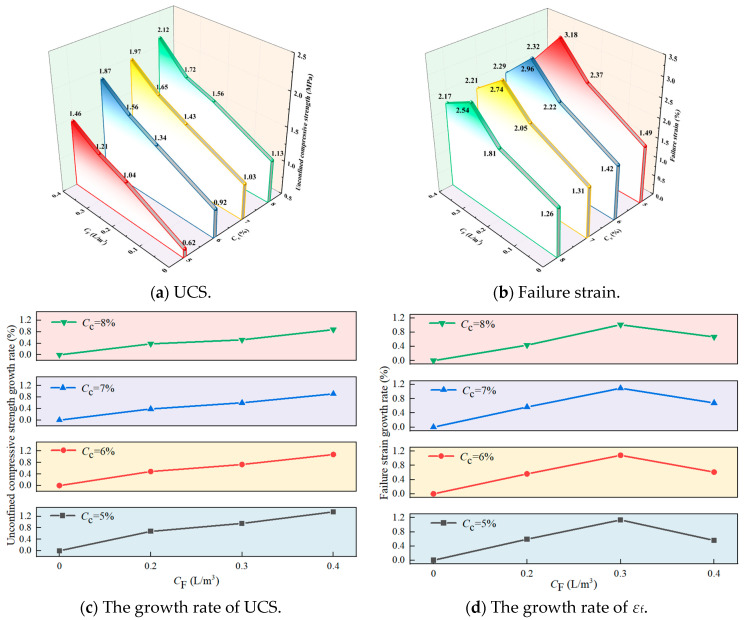
UCS, *ε*_f_, and UCS, and *ε*_f_ growth rate of FCIL with *C*_c_ and *C*_F_.

To investigate the effects of *C*_c_ and *C*_F_ on the UCS of FCIL and determine an appropriate mix proportion for FCIL, UC tests were conducted on loess specimens cured for 7 d under different conditions. Figure 2 shows the variations in UCS, *ε*_f_, the UCS increase ratio, and the *ε*_f_ change ratio of FCIL at different *C*_c_ and *C*_F_ levels (7 d curing), where the increase/change ratios are calculated relative to the cement-only group.

As can be seen from Figure 2a, when *C*_F_ = 0, the UCS increased from 0.62 MPa to 1.13 MPa as *C*_c_ increased from 5% to 8%, whereas *ε*_f_ decreased from 1.49% to 1.26%, indicating that cement can significantly improve the strength of loess, but cement-stabilized loess shows limited ductility and tends to fail in a brittle manner. Compared with cement-only stabilization, FCIL exhibited a better improvement performance at the same *C*_c_, achieving simultaneous increases in UCS and *ε*_f_. For example, when *C*_c_ = 6% and *C*_F_ = 0, the UCS and *ε*_f_ of FCIL were 0.90 MPa and 1.42%, respectively. When *C*_c_ = 6% and *C*_F_ = 0.2, 0.3, and 0.4 L/m^3^, the UCS of FCIL increased to 1.34, 1.56, and 1.87 MPa, corresponding to increases of 48.89%, 73.33%, and 108.11%, respectively; meanwhile, *ε*_f_ increased to 2.34%, 2.56%, and 2.29%, corresponding to increases of 56.28%, 108.38%, and 61.07%, respectively. Cement is the primary contributor to the strength gain of FCIL. By contrast, F1 increases the failure strain (εf) significantly while also enhancing strength, thereby improving the plasticity of FCIL. This improvement is mainly attributed to the ion-exchange effect of F1, which promotes flocculation and aggregation of soil particles. In addition, the combined action of F1 and cement hydration products forms a dense cemented skeleton. This skeleton fills interparticle pores and further increases the strength of loess. F1 can alleviate stress concentration induced by the rigid skeleton of cement hydration products, thus mitigating the high brittleness of cement-stabilized loess [27].

The 7 d UCS requirements for cement-stabilized materials used in highway pavement base and subbase layers are specified in the “Technical Guidelines for Construction of Highway Roadbases” [28]. Based on these requirements and the UCS results obtained in this study, FCIL is suitable for use as a base material for light- and medium-traffic Class II and lower highways. It can also be used as a subbase material for light-, medium-, and heavy-traffic Class II and lower highways. Considering both the specification requirements and cost-effectiveness, FCIL with *C*_c_ = 6% and *C*_F_ = 0.2 L/m^3^ provides the most favorable overall performance. This mix proportion not only meets the bearing capacity demand of the subbase but also retains a safety margin for subsequent strength development. In addition, its *ε*_f_ is 2.21%, which is significantly higher than that of loess treated with 6% cement alone (1.41%), effectively mitigating the brittleness of cement-treated loess and thereby reducing the risks of drying-shrinkage cracking and differential settlement over the full service life of the pavement structure.

### 3.2. Effect of C_c_ on FCIL Stress–Strain Relationship

**Figure 3:**
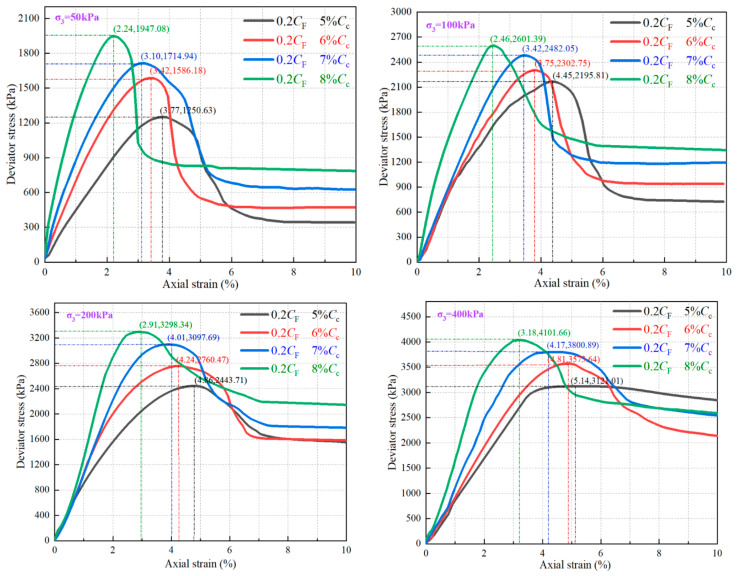
Stress–strain curves of FCIL under *C*_c_.

To analyze the effect of *C*_c_ levels on the triaxial stress–strain curves of FCIL, *C*_F_ was fixed at 0.2 L/m^3^, and the triaxial stress–strain curves of FCIL under four confining pressures (*σ*_3_ = 50, 100, 200, and 400 kPa) were plotted, as shown in Figure 3. The peak deviator stress on each triaxial stress–strain curve was taken as the failure point. For curves without an obvious peak, the deviator stress corresponding to an axial strain of 15% was used as the failure point [29]. The peak deviator stress was defined as the peak strength (*q*_max_), and the strain corresponding to *q*_max_ was defined as the failure strain (*ε*_f_). As shown in Figure 3, at different *σ*_3_ and *C*_c_ levels, the triaxial stress–strain curves of FCIL exhibited a typical strain-softening behavior. At a given *σ*3, as *C*_c_ increased, the stress–strain curves shifted overall to the left and upward, and the slope near the peak increased markedly, indicating that a higher *C*_c_ significantly increases *q*_max_ and the brittle behavior of FCIL. Taking *σ*_3_ = 200 kPa as an example, when *C*_c_ increased from 5% to 6%, 7%, and 8%, *q*_max_ of FCIL increased from 2443 kPa to 2760, 3097, and 3298 kPa, representing increases of 13%, 27%, and 35%, respectively. In contrast, *ε*_f_ decreased from 4.86% to 4.24%, 4.01%, and 2.91%, corresponding to reductions of 12.7%, 17.6%, and 40.1%, respectively.

Moreover, for FCIL with the same mix proportion, the stress–strain curves exhibited a sharp peak under low *σ*_3_, followed by a rapid post-peak stress drop; with increasing *σ*_3_, *q*_max_ continuously increased, the peak region gradually became flatter, and the residual strength (*τ*_r_) increased markedly. For FCIL with *C*_F_ = 0.2 L/m^3^ and *C*_c_ = 6%, under *σ*_3_ = 50, 100, 200, and 400 kPa, *q*_max_ was 1586.18, 2302, 2760, and 3573 kPa, respectively, whereas *τ*_r_ was 472, 938, 1585, and 2141 kPa, respectively. The *τ*_r_/*q*_max_ ratios were 29.77%, 40.78%, 57.44%, and 59.92%, respectively. These results indicate that, with increasing *σ*_3_, the strain-softening behavior of FCIL becomes less pronounced, and its post-failure stability and ductility are improved.

### 3.3. Effect of N_F-T_ on FCIL Stress–Strain Relationship

**Figure 4:**
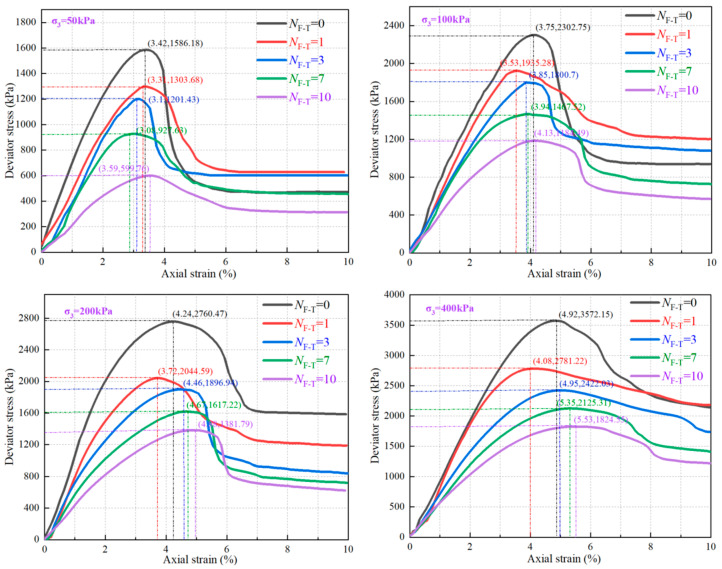
Stress–strain curve of FCIL under freeze–thaw cycles.

To investigate the effect of *N*_F-T_ on the triaxial stress–strain curves of FCIL, specimens with *C*_F_ = 0.2 L/m^3^ and *C*_c_ = 6% were selected. As shown in Figure 4, the triaxial stress–strain curves of FCIL under four confining pressures (50, 100, 200, and 400 kPa) were plotted. By comparing the curves under different conditions, all curves exhibited strain-softening behavior for different *N*_F-T_. At the same *σ*_3_, with increasing *N*_F-T_, the curves gradually became flatter, and *q*_max_ decreased. When *N*_F-T_ = 1, *q*_max_, *ε*_f_, and the initial slope of the curve all decreased significantly. As *N*_F-T_ increased to 3, 7, and 10, *q*_max_ continued to decrease, and τ_r_ decreased accordingly, whereas *ε*_f_ gradually increased and exceeded that in the *N*_F-T_ = 0. Taking *σ*_3_ = 200 kPa as an example, when *N*_F-T_ = 0, 1, 3, 7, and 10, *ε*_f_ was 4.24%, 3.72%, 4.46%, 4.67%, and 4.88%, respectively; compared with *N*_F-T_ = 0, the corresponding changes were −12.26%, 5.19%, 10.14%, and 15.09%, respectively. At *σ*_3_ = 50 kPa, when *N*_F-T_ = 0, 1, 3, 7, and 10, *q*_max_ was 1586, 1303, 1201, 927, and 599 kPa, respectively, corresponding to reductions of 18%, 24%, 41%, and 62% relative to *N*_F-T_ = 0. The corresponding τ_r_ values were 472, 627, 602, 457, and 313 kPa, respectively, and the τ_r_/*q*_max_ ratios were 29.87%, 48.15%, 49.33%, 50.12%, and 52.21% for *N*_F-T_ = 0, 1, 3, 7, and 10, respectively. These results indicate that the incorporation of F1 helps to suppress the abrupt post-failure strength drop of FCIL subjected to freeze–thaw cycling, enabling it to maintain relatively good structural stability and ductility during freeze–thaw deterioration.

### 3.4. Secant Deformation Modulus at 50% of Peak Strength

To evaluate the deformation resistance of FCIL under loading, the secant modulus corresponding to the deviator stress at 50% of *q*_max_ on the triaxial stress–strain curve was defined as *E*_50_ [30,31], as expressed in Equation (1):

(1) $$E_{50}=\frac{{\left(\sigma_{1}-\sigma_{3}\right)}_{f}}{2\cdot \varepsilon_{50\%}}$$

where *ε*_50%_ is the axial strain corresponding to 50% of *q*_max_.

**Figure 5:**
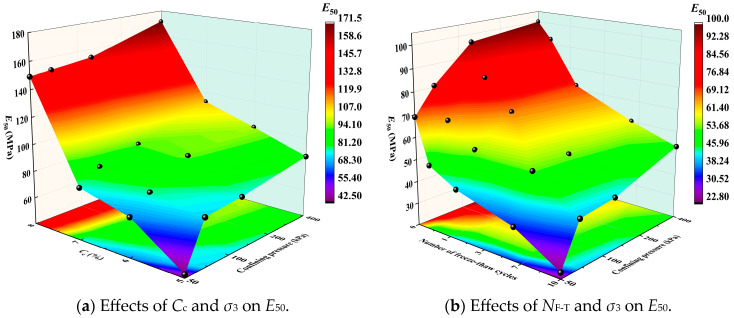
Variation in *E*_50_ of FCIL under different conditions.

The effects of *C*_c_ and *σ*_3_ on *E*_50_ of FCIL under different conditions are shown in Figure 5a. The results indicate that *E*_50_ increases markedly with increasing *C*_c_ and *σ*_3_. When *σ*_3_ = 50 kPa and *C*_c_ = 5%, 6%, 7%, and 8%, *E*_50_ was 42.7, 69.2, 78.4, and 149.1 MPa, respectively. Compared with *C*_c_ = 5%, *E*_50_ increased by 62%, 84%, and 249% at *C*_c_ = 6%, 7%, and 8%, respectively. Similarly, when *σ*_3_ = 200 kPa, the corresponding increases were 24.39%, 23.35%, and 98.58%, respectively. This indicates that, at a given confining pressure, increasing *C*_c_ can markedly increase *E*_50_. In addition, for the same mix proportion, *E*_50_ increased with *σ*_3_, whereas the rate of increase decreased as *C*_c_ increased. For example, when *C*_c_ = 5% and *σ*_3_ was 50, 100, 200, and 400 kPa, *E*_50_ was 42.7, 75.1, 77.5, and 86.3 MPa, respectively. Compared with *σ*_3_ = 50 kPa, *E*_50_ increased by 75.8%, 81.5%, and 102% at *σ*_3_ = 100, 200, and 400 kPa, respectively. In contrast, when *C*_c_ = 8%, *E*_50_ increased by only 1.14%, 3.21%, and 14.96% at *σ*_3_ = 100, 200, and 400 kPa, respectively. This suggests that, at low *C*_c_, the deformation resistance of the soil mainly depends on the lateral confinement provided by *σ*_3_. In contrast, at high *C*_c_, the reinforcement induced by the internal reactions of cement and F1 becomes dominant, thereby weakening the influence of *σ*_3_ on *E*_50_.

As shown in Figure 5b, for FCIL with *C*_F_ = 0.2 L/m^3^ and *C*_c_ = 6%, *E*_50_ decreases markedly with increasing *N*_F-T_, indicating that freeze–thaw cycling significantly weakens the deformation resistance of FCIL and makes it more prone to accumulated deformation under the same loading. When *σ*_3_ = 100 kPa and *N*_F-T_ = 0, 1, 3, 7, and 10, *E*_50_ of FCIL was 80.6, 66.5, 56.7, 54.7, and 41.0 MPa, respectively. Compared with *N*_F-T_ = 0, *E*_50_ decreased by 17%, 30%, 32%, and 49% at *N*_F-T_ = 1, 3, 7, and 10, respectively, and the reduction in deformation resistance was mainly concentrated within the first three freeze–thaw cycles. This is because freeze–thaw cycling generates microcracks due to ice expansion during the freezing stage, while water migration and particle erosion during the thawing stage further disturb the structure, thereby destroying the internal structure and aggregated state of FCIL; consequently, *E*_50_ decreases markedly with increasing number of freeze–thaw cycles [16,18]. Under low confining pressure, the lateral confinement of the soil is weak, and the microcracks generated during freeze–thaw cycling are more likely to propagate and coalesce, further reducing the deformation resistance. For example, when *σ*_3_ = 50 kPa, *E*_50_ at *N*_F-T_ = 10 was 22.8 MPa, which is 33% of that without freeze–thaw cycles. In contrast, when *σ*_3_ = 400 kPa, *E*_50_ at *N*_F-T_ = 10 was 53.1 MPa, accounting for 53.1% of that without freeze–thaw cycles. This indicates that high confining pressure helps to suppress the structural deterioration of the soil induced by freeze–thaw cycling.

### 3.5. Shear Strength Parameter on FCIL

**Figure 6:**
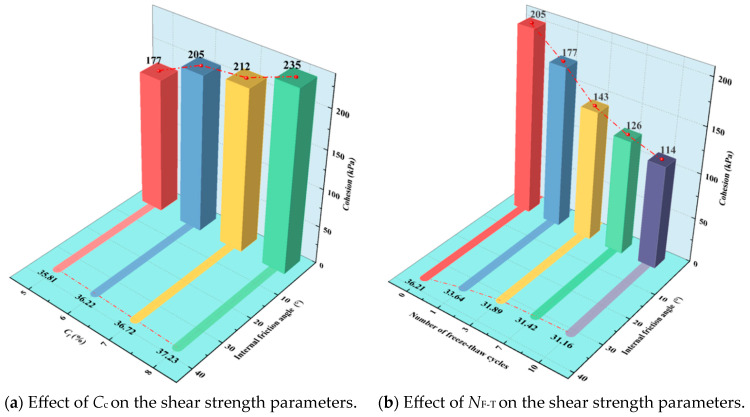
Different shear strength parameters of FCIL under various conditions.

To investigate the effects of *C*_c_ and *N*_F-T_ on the shear strength parameters of FCIL, a controlled-variable approach was adopted. As shown in Figure 6, the variations in the shear strength parameters of FCIL under different *C*_c_ and *N*_F-T_ conditions were compared and analyzed. When *C*_F_ = 0.2 L/m^3^, as *C*_c_ increased from 5% to 6%, 7%, and 8%, the *c* of FCIL increased from 177 kPa to 205, 212, and 235 kPa, corresponding to increases of 15.8%, 19.8%, and 32.7%, respectively. Meanwhile, *φ* increased from 35.8° to 36.2°, 36.7°, and 37.2°, representing increases of 1.11%, 2.57%, and 3.91%, respectively. The results indicate that increasing *C*_c_ can significantly increase *c* by enhancing interparticle cementation, whereas its effect on *φ*, which reflects interparticle friction, is relatively limited.

As shown in Figure 6b, for FCIL with *C*_F_ = 0.2 L/m^3^ and *C*_c_ = 6%, *c* decreased markedly with increasing *N*_F-T_, whereas *φ* remained nearly unchanged, which is consistent with the findings reported by Axel et al. [20]. When *N*_F-T_ = 1, 3, 7, and 10, *c* and *φ* decreased (relative to *N*_F-T_ = 0) to 177, 143, 126, and 114 kPa, and to 33.6°, 31.9°, 31.4°, and 31.1°, respectively, corresponding to reductions of 13.66%, 30.24%, 38.54%, and 44.39% for c, and 7.07%, 11.91%, 13.2%, and 13.92% for *φ*; the deterioration was most pronounced during the first and early freeze–thaw cycles. During freeze–thaw cycling, freeze-induced expansion and thaw-related leaching cause repeated volumetric changes in internal pores, which disrupt the cemented structure of FCIL and the interparticle bonding interfaces, thereby significantly reducing *c*. In contrast, *φ* is mainly governed by interparticle friction and interlocking, and is therefore less affected by freeze–thaw cycling.

### 3.6. Failure Characteristics of FCIL

**Figure 7:**
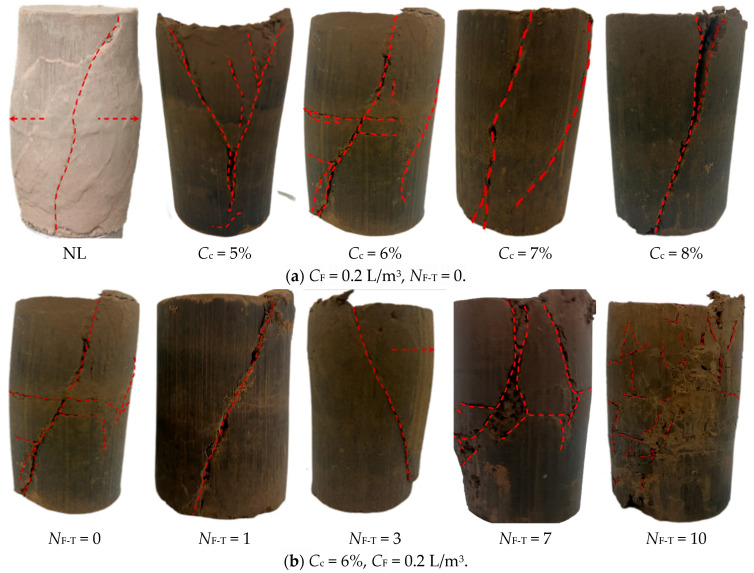
The failure of CU test specimens of improved loess.

To visually examine the failure modes of FCIL specimens under triaxial shearing and their dependence on *C*_c_ and *N*_F-T_, the triaxial shear failure patterns of different soils were investigated, as shown in Figure 7. As shown in Figure 7a, the natural loess specimen developed a single major shear band at failure. Macroscopically, an inclined shear crack extended from the bottom to the top, accompanied by pronounced bulging deformation, indicating an overall plastic failure mode. With increasing *C*_c_, the number of failure cracks in FCIL specimens decreased markedly, and the failure mode gradually transitioned from multi-crack shearing to a dominant single inclined shear plane. This suggests that higher *C*_c_ increases the stiffness of FCIL specimens, leading to a brittle failure behavior.

As shown in Figure 7b, *N*_F-T_ had a significant influence on the failure mode of FCIL specimens. When *N*_F-T_ ≤ 3, FCIL specimens failed along a single inclined shear plane. The failure process was characterized by sudden brittle failure: the shear band formed rapidly and propagated through the specimen within a short time. Bulging deformation was not pronounced, indicating a predominantly brittle response with limited ductile deformation and poor structural integrity. When *N*_F-T_ = 7 and 10, numerous intersecting cracks developed on the lateral surface and the top of the FCIL specimens. The single shear plane transitioned into a fragmented shear zone, and bulging became markedly more pronounced. In addition, *ε*_f_ was greater than that of the specimens without freeze–thaw cycles. The stress–strain curves exhibited a pronounced reduction in peak strength and a relatively flattened post-peak response. This can be attributed to the fact that, during freeze–thaw cycling, the expansion of pore water upon freezing compresses soil particles and hydration products and weakens interparticle cementation. During thawing, the melting and shrinkage of ice crystals transform the original ice-occupied zones into pores and promote an increase in pore size [23]. As a result, FCIL shifts from a high-strength, brittle shear failure in the early cycles to a plastic failure mode with reduced strength and enhanced ductility [32].

### 3.7. Microstructural Characteristics and Synergistic Improvement Mechanism Analysis

#### 3.7.1. Pore Characteristics of the FCIL

Following the pore classification method for soils [18], the pores in FCIL were divided into four categories: macropores (>1 μm), mesopores (0.1–1 μm), small pores (0.1–0.01 μm), and micropores (<0.01 μm). The effects of *C*_c_ and *N*_F-T_ on the distribution characteristics of the micropore structure of FCIL were analyzed.

(1) Effect of *C*_c_ on the pore characteristics of FCIL

**Figure 8:**
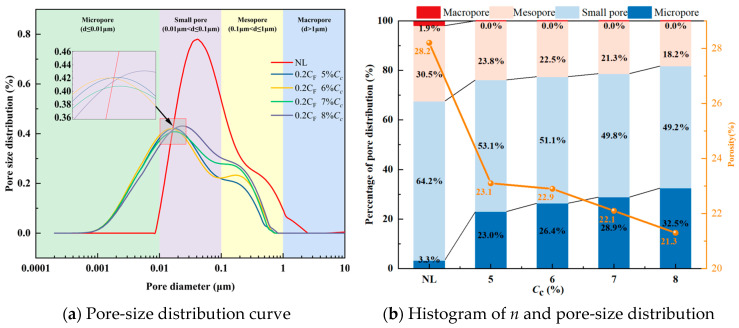
Pore characteristics of FCIL at different *C*_c_.

Based on NMR results, the pore-size distribution curves of FCIL at different *C*_c_ were plotted, as shown in Figure 8a. The results indicate that the pore-size distribution curve of NL is mainly concentrated in the ranges of small and medium pores, suggesting that the pore structure is dominated by small and medium pores, with a considerable number of connected macropores. When *C*_F_ = 0.2 L/m^3^, as *C*_c_ increased, the peak of the pore-size distribution curve of FCIL decreased significantly, and the curve shifted overall to the right, indicating that after stabilization, the contents of small and medium pores in loess decreased markedly and evolved toward micropores, while large pores were significantly reduced and even almost disappeared.

The variations in *n* and the pore-size distribution percentages in FCIL at different *C*_c_ values are shown in Figure 8b. The results indicate that, with increasing *C*_c_, *n*, and the proportions of medium and small pores in FCIL decreased markedly, macropores disappeared, and micropores increased. Compared with NL, when *C*_c_ was 5%, 6%, 7%, and 8%, the *n* value of FCIL decreased by 17.73%, 20.97%, 23.63%, and 26.60%, respectively. The proportions of medium pores decreased by 24.19%, 28.33%, 32.15%, and 41.98%, and those of small pores decreased by 19.76%, 22.92%, 24.82%, and 25.68%, respectively, whereas the proportion of micropores increased by 567.25%, 664.06%, 736.52%, and 842.61%, respectively. This can be attributed to the ion-exchange effect of F1, which disrupts the electric double-layer water film structure, alters the contact and arrangement of particles, and thereby makes the soil denser [11,12]. Meanwhile, cement generates cementitious gels, such as C-S-H and C-A-H, as well as crystalline products such as AFt through hydration, which fill interparticle pores and bond soil particles. While reducing the total pore volume, cement also promotes the transformation of medium and large pores into small and micropores, thereby significantly increasing soil strength.

(2) The effect of *N*_F-T_ on the pore characteristics of FCIL

**Figure 9:**
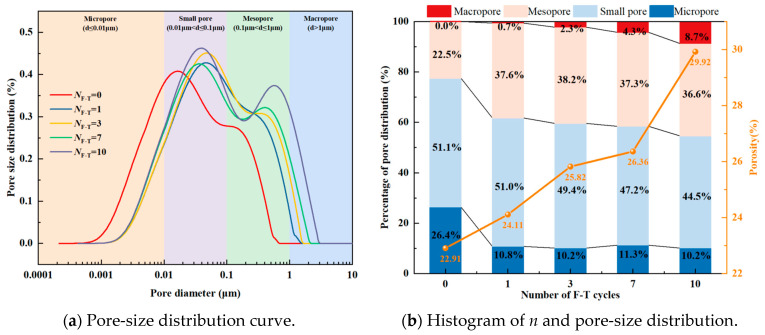
Pore characteristics of FCIL at different *N*_F-T_.

Based on the NMR test results, the pore-size distribution curves of FCIL under different *N*_F-T_ at *C*_F_ = 0.2 L/m^3^ and *C*_c_ = 6% are shown in Figure 9a. It can be seen that when *N*_F-T_ = 0, the pore-size distribution is a unimodal distribution with a relatively reasonable pore-size allocation. With increasing *N*_F-T_, the pore-size distribution curves shift upward and to the right overall, and gradually become bimodal, with increased proportions of medium and large pores. This indicates that freeze–thaw cycling not only markedly increases *n* but also shifts the pore-size distribution toward larger pores.

The variations in *n* and the pore-size distribution proportions of FCIL with *N*_F-T_ are shown in Figure 9b. The results indicate that, with increasing *N*_F-T_, *n* as well as the proportions of large and medium pores increased significantly, whereas the proportions of small pores and micropores decreased. Compared with *N*_F-T_ = 0, when *N*_F-T_ = 1, 3, 7, and 10, *n* increased by 5.24%, 12.7%, 15.06%, and 30.6%, respectively; the proportion of large pores increased by 0.66%, 2.25%, 4.29%, and 8.72%, respectively; the proportion of medium pores increased by 66.7%, 69.4%, 65.41%, and 62.66%, respectively. In contrast, the proportion of small pores decreased by 0.27%, 3.16%, 7.66%, and 12.89%, respectively, and the proportion of micropores decreased by 57.25%, 59.10%, 61.49%, and 61.46%, respectively. With increasing *N*_F-T_, repeated frost heave due to pore-water freezing induces cracking of the original cemented structure and promotes the propagation and coalescence of microcracks, thereby damaging the dense small-pore structure and particle aggregates. Consequently, small pores and micropores gradually coarsen into medium and large pores, manifested by a notable increase in n and the proportion of large pores, a notable increase in the proportion of medium pores, and decreases in the proportions of small pores and micropores.

#### 3.7.2. Mechanism Analysis of FCIL Under Freeze–Thaw Cycling

**Figure 10:**
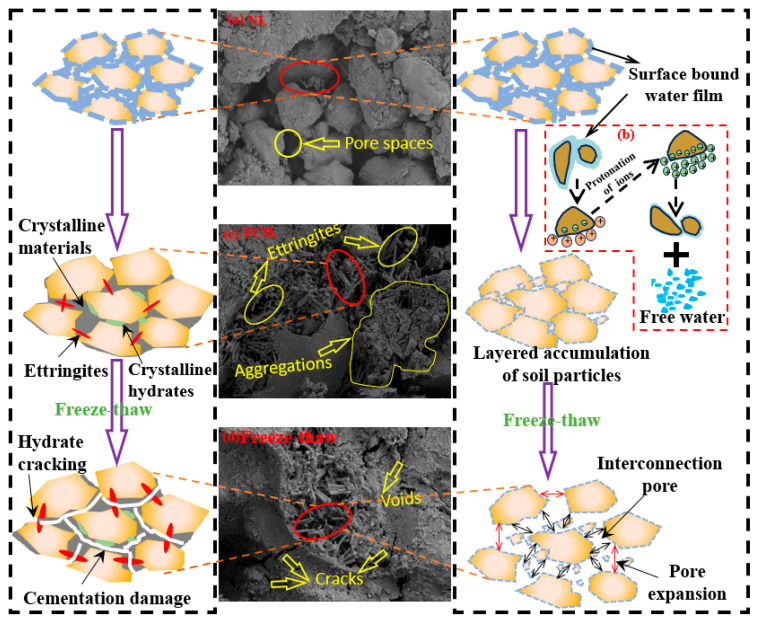
Schematic diagram of reinforcement and freeze–thaw damage mechanism of FCIL. (**a**) Loess; (**b**) Mechanism of F1 action; (**c**) FCIL; (**d**) Freeze-thaw. The SEM images were obtained at 5.00 kV using the SE2 signal detector; the working distance was 8.9 mm and the magnification was 5.00 KX.

Figure 10a shows that NL particles exhibit irregular shapes with a loose arrangement and a complex pore structure [33]. As shown in Figure 10b, upon F1 incorporation, ion-exchange reactions occur, which disrupt the electric double-layer structure and replace weakly bound cations (e.g., Ca^2+^ and Mg^2+^) as well as part of the polar water molecules, thereby effectively reducing the thickness of the bound-water film and the interparticle spacing and promoting flocculation and aggregation of soil particles [26]. As shown in Figure 10c, when cement is added, hydration reactions take place, producing C-S-H and C-A-H gels and AFt crystals, which grow and interweave between soil particles, fill pores, and bond the particles. Under compaction, a dense stacked structure is formed, thus significantly improving the soil strength.

Figure 10d shows that, during freeze–thaw cycling, the freezing expansion and thaw-induced migration of internal water repeatedly compress and erode the soil skeleton, damaging the cementation among loess particles, increasing the number of cracks and pores, and significantly reducing soil strength. With the incorporation of F1, ion-exchange reactions can disrupt the electric double-layer water film structure on the particle surfaces [34]. In addition, the sulfonated oil component in F1 can form a hydrophobic layer on the particle surfaces, thereby enhancing the hydrophobicity of soil particles. This markedly weakens capillary water transport between particles and reduces the driving force for water migration during freeze–thaw cycling. It also reduces repeated compression of the soil skeleton caused by volumetric changes associated with pore-water phase transitions, mitigates dissolution and erosion after thawing, and inhibits microcrack initiation and propagation [35]. Consequently, the cumulative structural damage induced by freeze–thaw cycling is mitigated, thereby improving the stability and durability of FCIL in freeze–thaw environments.

## 4. Conclusions

In this study, cement and F1 were used synergistically to reinforce loess. Through a series of macroscopic mechanical tests and microstructural characterizations, the effects of *C*_c_, *C*_F_, and *N*_F-T_ on the stress–strain curves, *q*_max_, shear strength parameters, failure characteristics, and pore-size distributions of improved loess were investigated. The improvement mechanism of FCIL, as well as the freeze–thaw damage mechanism, was clarified. Based on the experimental results, the following conclusions can be drawn:

(1) The cement content is the primary factor governing the strength of improved loess, while the incorporation of F1 can synergistically enhance strength and markedly increase the failure strain, thereby achieving a simultaneous optimization of strength and ductility. Based on the UC test results, the optimal mix proportion of FCIL was determined as *C*_c_ = 6% and *C*_F_ = 0.2 L/m^3^. After 7 days of curing, the UCS and *ε*_f_ of FCIL reached 1.35 MPa and 2.21%, respectively. This mix proportion not only satisfies the strength requirements for subgrade materials but also effectively reduces cement consumption and alleviates the brittleness of cement-improved loess, demonstrating good engineering and economic benefits.
(2) Compared with *C*_F_ = 0 L/m^3^ (*C*_c_ = 6%), when *C*_F_ = 0.2, 0.3, and 0.4 L/m^3^, the UCS of FCIL increased by 48.89%, 73.33%, and 108.11%, respectively, and the corresponding *ε*_f_ increased by 56.28%, 108.38%, and 61.07%, respectively. With increasing *C*_c_, enhanced interparticle cementation significantly increases the *c* of FCIL, resulting in higher stiffness and a more brittle failure behavior of the specimens, whereas the increase in *φ* is relatively limited. With increasing *N*_F-T_, numerous intersecting cracks develop on the lateral surface and top of the specimens; the single shear plane gradually transitions into a crushed shear band, and the bulging phenomenon becomes more pronounced. Accordingly, the stress–strain curves exhibit a plastic failure feature characterized by a marked decrease in *q*_max_ and a flatter post-peak response.
(3) NMR and SEM analyses indicate that, after improvement with cement and F1, the proportions of small and medium pores, as well as the *n*, decreased significantly, whereas micropores increased and large pores almost disappeared. With increasing *N*_F-T_, the proportions of medium and large pores and *n* increased markedly, while small pores and micropores decreased, indicating that the pore-size distribution evolved toward medium and large pores. F1 can thin the water film and reduce interparticle spacing through ion exchange. Cement hydration products can bond soil particles and fill interparticle pores, while the sulfonated oil component of F1 forms a hydrophobic barrier that suppresses water migration and microcrack propagation. Consequently, the pore structure of loess is substantially improved, leading to enhanced strength and improved stability against freeze–thaw cycling.

## Figures and Tables

**Table 1:** Physical and mechanical parameters of test materials.

NL	F1	Cement
Natural density: 1.39 g/cm^3^	Density: 1.34 g/cm^3^	Strength grade: 42.5
Liquid limit: 26.3%	Water solubility: 100%	Density: 3.12 g/cm^3^
Plastic limit: 18.86%	Acidity: 0.5~1.5	Initial setting time: 261 min
Optimal water content: 14.64%	Volatilization rate: <1%	Final setting time: 312 min
Maximum dry density: 1.787 g/cm^3^	Boiling point: >100 °C	Compressive strength (3/28 d): 20/40.1 MPa
Plasticity index: 7.44	Solidifying point: <0 °C	Flexural strength (3/28 d): 4.3/7.2 MPa

**Table 2:** Test parameters and working condition settings.

	*N*_F-T_	*σ*_3_ (kPa)	*C*_F_ (L/m^3^)	*C*_c_ (%)	*v* (mm/min)
UC tests	0		0, 0.2, 0.3, 0.4	5, 6, 7, 8	1
CU tests	0	50, 100, 200, 400	0.2	5, 6, 7, 8	0.5
	1, 3, 7, 10	50, 100, 200, 400	0.2	6	0.5
NMR	0		0, 0.2	0, 5, 6, 7, 8	
	1, 3, 7, 10		0.2	6	
SEM tests	0, 10		0, 0.2	0, 6	

## Data Availability

The original contributions presented in this study are included in the article. Further inquiries can be directed to the corresponding authors.
